# Exergy Analysis of Two-Stage Organic Rankine Cycle Power Generation System

**DOI:** 10.3390/e23010043

**Published:** 2020-12-30

**Authors:** Guanglin Liu, Qingyang Wang, Jinliang Xu, Zheng Miao

**Affiliations:** Beijing Key Laboratory of Multiphase Flow and Heat Transfer for Low Grade Energy Utilization, North China Electric Power University, Beijing 102206, China; liu0513@126.com (G.L.); wangqy@ncepu.edu.cn (Q.W.); xjl@ncepu.edu.cn (J.X.)

**Keywords:** two-stage power generation system, split ratio, organic Rankine cycle, exergy analysis, organic working medium

## Abstract

Organic Rankine cycle (ORC) power generation is an effective way to convert medium and low temperature heat into high-grade electricity. In this paper, the subcritical saturated organic Rankine cycle system with a heat source temperature of 100~150 °C is studied with four different organic working fluids. The variations of the exergy efficiencies for the single-stage/two-stage systems, heaters, and condensers with the heat source temperature are analyzed. Based on the condition when the exergy efficiency is maximized for the two-stage system, the effects of the mass split ratio of the geothermal fluid flowing into the preheaters and the exergy efficiency of the heater are studied. The main conclusions include: The exergy efficiency of the two-stage system is affected by the evaporation temperatures of the organic working fluid in both the high temperature and low temperature cycles and has a maximum value. Under the same heat sink and heat source parameters, the exergy efficiency of the two-stage system is larger than that of the single-stage system. For example, when the heat source temperature is 130 °C, the exergy efficiency of the two-stage system is increased by 9.4% compared with the single-stage system. For the two-stage system, analysis of the four organic working fluids shows that R600a has the highest exergy efficiency, although R600a is only suitable for heat source temperature below 140 °C, while other working fluids can be used in systems with higher heat source temperatures. The mass split ratio of the fluid in the preheaters of the two-stage system depends on the working fluid and the heat source temperature. As the heat source temperature increases, the range of the split ratio becomes narrower, and the curves are in the shape of an isosceles triangle. Therefore, different working fluids are suitable for different heat source temperatures, and appropriate working fluid and split ratio should be determined based on the heat source parameters.

## 1. Introduction

Due to the increasingly prominent environmental problems and fossil energy depletion, using renewable energy such as geothermal energy, solar energy and wind energy and industrial waste heat for power generation is of increasing interest. Compared to solar and wind energy, geothermal energy has the advantage of stable temperature, and organic Rankine cycle (ORC) power generation system using medium and low temperature geothermal energy has become an intense field research. With different parameters, such as heat source temperature and working fluid, the thermal efficiency of the system varies greatly [1]. Optimization of the system under different parameters for higher system efficiency is mainly based on the first law of thermodynamics.

The advantage of the second law of thermodynamics (exergy analysis) over the first law is that it can identify the key parts to improve the efficiency of the system. However, the second law of thermodynamics is seldomly studied on ORC power generation systems, and current research is mainly focused on simple system thermodynamic parameters, core equipment exergy losses and combined systems. For example, in terms of system parameters and working fluid analysis, Yildiz et al. [2] analyzed the operating pressure, temperature and other parameters of the ORC system using waste heat from exhaust gas. They compared the subcritical and supercritical cycles, and obtained higher system performance during subcritical operation. Sun et al. [3] studied ORC system driven by industrial low-temperature waste heat, and it is found that the evaporation temperature of working medium has the greatest influence on the exergy efficiency of the system. Under a certain heat source temperature, the exergy efficiency of the system first increases and then decreases with the increase of the heat source temperature. Altun et al. [4] analyzed the net power generation and exergy efficiency of geothermal ORC power station under different ambient temperatures. It is found that the temperature in different seasons has great influence on the net power generation, and the re-injection exergy loss is the largest part. It is proposed that an internal heat recovery system can be used to improve the system efficiency. Junhu et al. [5] and Shuaijie et al. [6] analyzed the optimal evaporation temperatures for more than 10 working fluids in the subcritical system and studied their variations. They found that each working fluid has an optimal evaporation temperature which maximizes the net output of the system. Research on the selection of working fluid was also performed and it was found that a certain relationship exists between the critical temperature of the working fluid and the thermal efficiency of the system [7,8]. Five types of dry working fluids were studied to analyze the variation of the low-temperature ORC system under different control parameters, and it was found that when the working fluid is R600a, the system thermal efficiency, exergy efficiency and other parameters are optimized [9]. The performance of the subcritical saturated ORC system using six azeotropic mixtures was studied, and it was found that the exergy efficiency of the system when the working fluid is mixed is higher than that when pure working fluid is used [10].

The exergy efficiency analysis of key components can clearly provide the direction of system optimization. By analyzing the exergy efficiency of key equipment under different heat source temperatures, working fluids and other parameters, it was found that the evaporator had the largest exergy loss among all component [2,6,9,11,12], indicating that improving the heat transfer performance of the evaporator is an effective way to improve system efficiency. Comparing the operating parameters of different systems, it was found that the evaporation pressure has a significant effect on the thermal efficiency and exergy efficiency [13,14]. Based on an exergy analysis for a simple ORC system, it was found that as the temperature difference at the pinch point of the evaporator increases, the system exergy efficiency decreases [15].

In terms of power generation system research, the exergy efficiency of coupled absorption refrigeration cycle and ejector refrigeration cycle was analyzed, and it was found that as the ORC evaporation temperature increases, the exergy efficiency of both systems decreases [3]. Analyzing the low-temperature geothermal ORC series-parallel system and the cogeneration system, it was found that when working fluid with higher critical parameter is used, the series ORC system has higher efficiency, while the parallel system is suitable for working fluid with lower critical temperature [16]. By comparing the efficiency of the ORC system with different numbers of regenerators, it was found that when the critical pressure of the working fluid is small, the exergy efficiency of the system is low [17]. An exergy analysis of three kinds of high-temperature geothermal ORC systems was carried out, and it was proposed that it is advantageous to include a heat recovery and regeneration system [18]. Analyzing the subcritical ORC cogeneration system driven by solar energy and biogas boiler, it was found that the net power generation efficiency of the cogeneration system is higher than that of the basic system [19].

The two-stage ORC system can realize the cascade utilization of energy and improve the energy conversion efficiency, but they are seldomly studied. Studies on the low-temperature heat source two-stage ORC power generation system found that the performance of the two-stage ORC system is better than that of the single-stage ORC system. Exergy analysis found that the high pressure evaporator had the largest exergy loss [20,21]. Exergy topology method was used to study the exergy flow and loss of ORC system, and the influence of pinch temperature difference on system performance was analyzed, and it was found that the smaller point temperature difference small, the system exergy efficiency is higher [22].

The above discussion shows that there are few studies on the two-stage ORC power generation system, and the influence of heat source temperature, working fluid and other parameters on the system needs to be further studied. Moreover, research on the preheater shunt has not been reported. In this work, the EES software is used to study the two-stage ORC power generation system. Based on the principle of energy cascade utilization, the thermal fluid cooling process and the working fluid heating-evaporation process are better matched, and the system efficiency can be improved theoretically. On this basis, the variation of the split ratio in the two-stage system is analyzed.

## 2. Power Generation System and Calculation Model

### 2.1. Power Generation System

**Figure 1:**
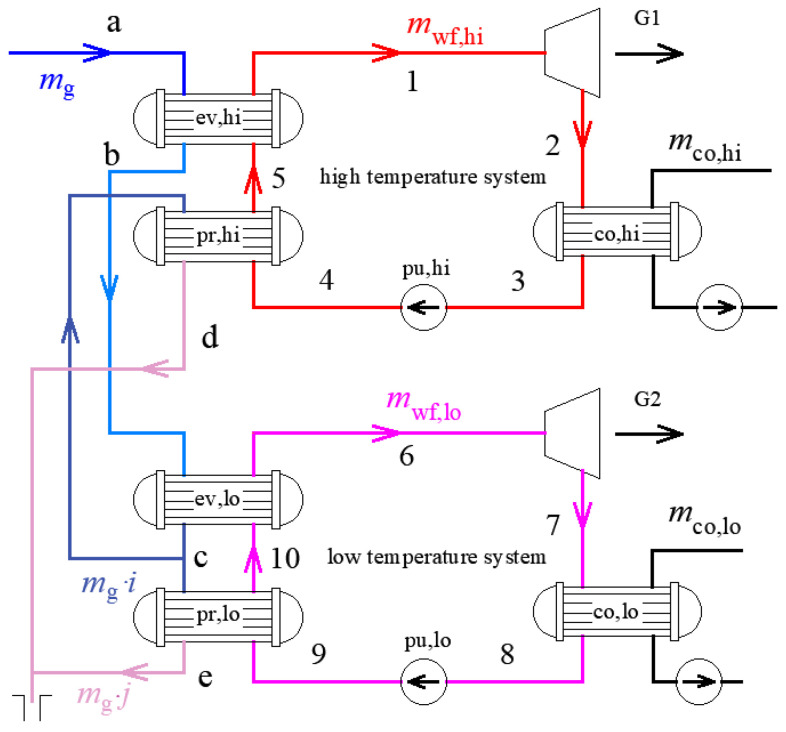
Schematic diagram of the two-stage organic Rankine cycle (ORC) system.

**Figure 2:**
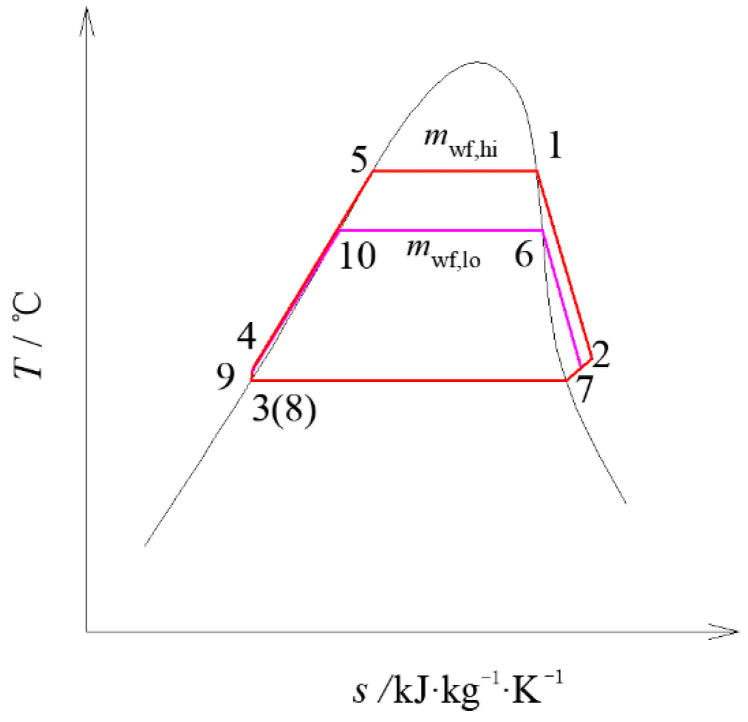
Temperature–entropy (*T*–*s*) diagram of the two-stage ORC system.

Compared with single system, the two-stage cycle power generation system has relatively independent high-temperature and low-temperature systems. Figure 1 shows the flow chart of the two-stage ORC power generation system. Figure 2 shows the system T-s diagram corresponding to Figure 1. The entire system mainly includes the geothermal fluid circulation in the heat source part and the organic working fluid circulation for power generation.

The high pressure geothermal fluid circulation, which is liquid in the heat transfer process, has the following procedure: the geothermal fluid enters the high temperature evaporator (ev,hi) of the high temperature ORC system from the geothermal well, exchanges heat with the organic working fluid, and then enters the low temperature evaporator (ev,lo) of the low temperature ORC system, and exchanges heat with organic working fluid. Subsequently, the geothermal fluid is divided into two streams, entering the preheaters (pr,hi and pr,lo) of the high and low temperature systems respectively, preheating the organic working fluid separately, and finally being injected into the recharge well.

The power generation system using organic working fluid is a subcritical saturated system, including a high temperature system and a low temperature system. The two systems have the same working principle, and the high temperature system is taken as an example and discussed below. The liquid organic working fluid is pressurized by the pump (pu,hi) and then enters the preheater (pr,hi) and the evaporator (ev,hi) to exchange heat with the geothermal fluid. In this process, the organic working fluid is heated from sub-cooled liquid to saturated gas, then enters the expander and expands to do work. The exhaust steam is discharged from the expander and enters the condenser (co,hi) to be condensed into liquid, completing the closed-loop cycle. The regenerative power generation system is added a heat exchanger at the expander outlet to exchange heat between the expander outlet and the pump outlet fluid, so as to improve the energy utilization.

To eliminate the ozone destruction and greenhouse effects, working fluids that are environmentally friendly are proposed to be used. In this paper, the selected working fluids include hydrocarbons (HCs) and hydrofluorocarbons (HFCs), and the flammability, toxicity, chemical characteristics and other physical properties of the working fluid are comprehensively considered. By comparing the working fluids used in the literature, the organic working fluids shown in Table 1 are selected for analysis.

### 2.2. Mathematical Model

The net output electric power of a power generation system is defined as the expander output electric power minus the working fluid pumping power consumption. The calculation method of the two-stage ORC power generation system is the same. Taking the high temperature system as an example, the net output electric power calculation formulas are listed as follows:

*W*_net,hi_ = *W*_exp,hi_ − *W*_pu,hi_ (1)

*W*_exp,hi_ = *m*_wf,hi_ (*h*_1_ − *h*_2_) (2)

*W*_pu,hi_ = *m*_wf,hi_∙*v*_3_∙(*p*_1_ − *p*_2_)/*η*_pu_ (3)

It is assumed that the geothermal fluid is incompressible, and the flow rate of organic working fluid in the high temperature system (*m*_wf,hi_) is:

(4) $$m_{\mathrm{wf},\mathrm{hi}}={\bar{c}}_{p}\cdot m_{g}\cdot (t_{a}\text{-}t_{\mathrm{ev},\mathrm{hi}}-\Delta t_{\mathrm{hi}})/\left(h_{1}-h_{5}\right)$$

The system’s exergy efficiency is calculated as:

(5) $$\eta_{\mathrm{ex}}\;=\;\frac{W_{\mathrm{net},\mathrm{hi}}}{m_{g}\cdot [\left(h_{a\;}{-\;h}_{0}\right){\;-\;T}_{0}\cdot \left(s_{a}\;-\;s_{0}\right)]}$$

The effective exergy of the heater (including evaporator and preheater) in the high temperature system can be calculated as:

*EX*_(*ev*+ph)ga,hi_ = *m*_wf,hi_∙[*h*_1_ − *h*_4_ − *T*_0_∙(*s*_1_ − *s*_4_)] (6)

The consumed exergy in the evaporator and preheater can be calculated as:

*EX*_(*ev*+ph)pa,hi_ = *m*_g_∙[*h*_a_ − *h*_b_ − *T*_0_∙(*s*_a_ − *s*_b_)] + *i*∙[*h*_c_ − *h*_d_ − *T*_0_∙(*s*_c_ − *s*_d_)] (7)

The exergy efficiency of the heater is then expressed as:

(8) $$\eta_{\left(ev+\mathrm{ph}\right)\;\mathrm{ex},\mathrm{hi}}\;=\;\frac{{EX}_{\left(ev+\mathrm{ph}\right)\mathrm{ga},\mathrm{hi}}}{{EX}_{\left(ev+\mathrm{ph}\right)\mathrm{pa},\mathrm{hi}}}$$

The effective exergy of the condenser in the high temperature system can be calculated as

*EX*_co,ga,hi_ = *m*_cw,hi_∙[(*h*_cw,out_ − *h*_co,in_) − *T*_0_∙(*s*_cw,out_ − *s*_co,in_)] (9)

The consumed exergy in the condenser is:

*EX*_co,pa,hi_ = *m*_wf,hi_∙[(*h*_2_ − *h*_3_) − *T*_0_∙(*s*_2_ − *s*_3_)] (10)

The exergy efficiency of the condenser is expressed as:

(11) $$\eta_{\mathrm{co},\mathrm{ex},\mathrm{hi}}\;=\;\frac{{EX}_{\mathrm{co},\mathrm{ga},\mathrm{hi}}}{{EX}_{\mathrm{co},\mathrm{pa},\mathrm{hi}}}$$

In the two-cycle geothermal fluid power generation system, the geothermal fluid exits evaporator of the low temperature system and enters the preheaters of the high temperature and low temperature systems. The split ratio of the geothermal fluid affects the energy exchange and efficiency in the preheater. Therefore, the flow rate of the geothermal fluid needs to be properly divided to meet the maximum effective use of energy, thereby reducing equipment size. The split ratio meets the following conditions:

(12) $$i=m_{\mathrm{wf}.\mathrm{hi}}\cdot \left(h_{5}-h_{4}\right)/{\bar{c}}_{p}\cdot \left(t_{c}-t_{d}\right)\cdot m_{g}$$

(13) $$j=m_{\mathrm{wf},\mathrm{lo}}\cdot \left(h_{10}-h_{9}\right)/{\bar{c}}_{p}\cdot \left(t_{c}-t_{e}\right)\cdot m_{g}$$

where *h*, *p*, *v*, *s* are the enthalpy, pressure, mass volume and entropy of the working fluids, the numbers and letters in the subscripts correspond to the points in Figure 2, and *x* and *y* represent inlet and outlet conditions of the cooling water. *η*_p_ is the working fluid pump efficiency, ${\bar{c}}_{p}$ is the average specific heat of the geothermal fluid, *m*_g_ is the mass flow rate of the geothermal fluid, *m*_cw,hi_ is the cooling water flow rate, *i* is the flow rate ratio of the geothermal fluid entering high temperature preheater after splitting, *t*_a_ is the initial temperature of geothermal fluid, *t*_ev,hi_ is the evaporation temperature of organic working fluid in high temperature system, Δ*t*_hi_ and Δ*t*_lo_ are the pinch temperature of evaporator in high temperature and low temperature systems, respectively, *T*_0_ is the environmental temperature, *t*_3_ and *t*_7_ is the condensation temperature of organic working fluid. The influence of impurity, non-condensable gas in geothermal fluid and ORC pressure drop is ignored, and the main parameters are shown in Table 2.

## 3. Results and Discussion

### 3.1. Exergy Analysis of Systems and Components

To study the subcritical saturated power generation system driven by the medium and low temperature geothermal fluid, the exergy efficiencies of the single-stage system, regenerator system and two-stage system controlled by multiple parameters are analyzed and compared first. The exergy efficiencies of heater (evaporator and preheater) and condenser are then analyzed. On this basis, variation of the split ratio of geothermal fluid and the exergy efficiency of the heater in the two working fluid power generation system are analyzed.

The exergy efficiency of the system at saturation temperature is calculated, so the temperature of working fluid at the outlet of evaporator is saturation temperature, corresponding to a certain pressure. The temperature of the working fluid at the outlet of condenser is 30 °C, corresponding to the saturation pressure at this temperature. Due to the difference of physical properties of working fluids, the corresponding pressure is different at the same temperature.

**Figure 3:**
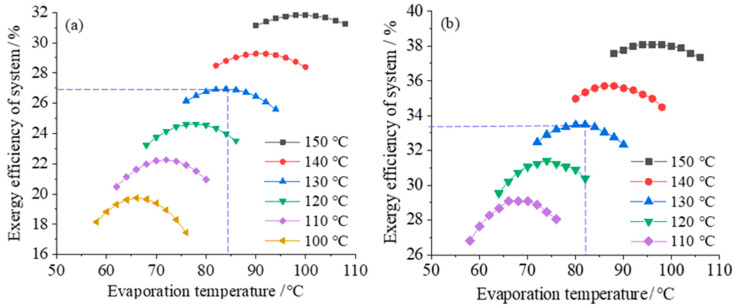
Exergy efficiency varies with evaporation temperature: (**a**) for the single-stage ORC system, (**b**) for the regeneration ORC system.

First, the exergy efficiencies of the two-stage and single-stage power generation systems are analyzed and compared, and the working fluid is R600a. Figure 3 shows the variation of the exergy efficiency of the system with the evaporation temperature of the organic working fluid in the evaporator when the heat source temperature is different. It can be seen from the Figure 3 that as the temperature of the heat source increases, the overall exergy efficiency of the system shows an increasing trend. When the temperature of the heat source is constant and the evaporation temperature of the working fluid increases, the exergy efficiency of the system first increases and then decreases, that is, there is a maximum value for the exergy efficiency of the system. Sun et al. [3] studied the ORC system and found that the exergy efficiency of the system first increases and then decreases with the increase of the heat source. Our conclusions are consistent with the results of Sun et al. [3].

It can be seen from Figure 3a, when the temperature of the heat source is 130 °C, the maximum exergy efficiency of the system is 26.9% for the single-stage ORC system, and the exergy efficiency of regeneration ORC system is 33.2% at the same heat source temperature, which shows in Figure 3b. The exergy efficiency of the regenerative system is high. The above results are consistent with those of Altun et al. [4].

**Figure 4:**
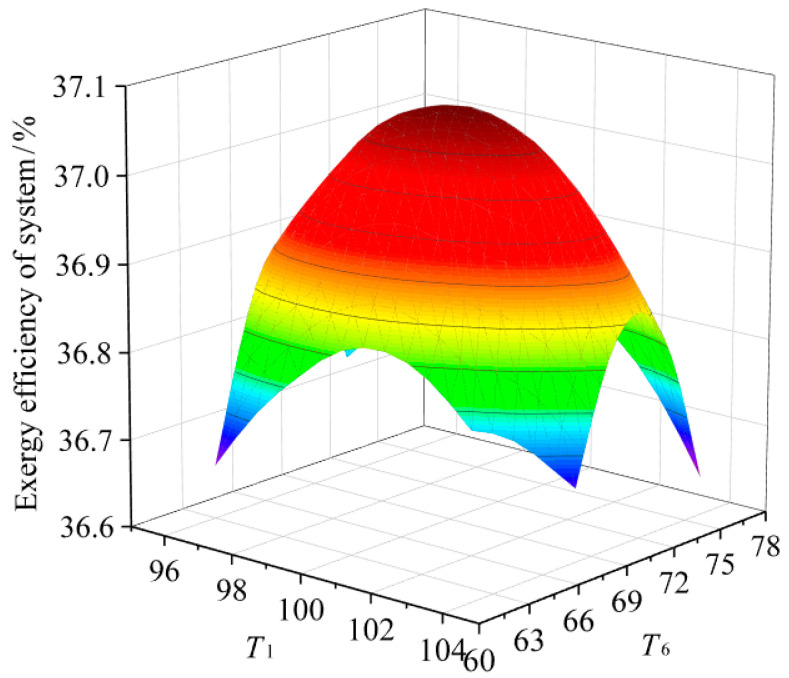
Relationship between the efficiency of two-stage system and the evaporative temperature in high and low temperature system.

Figure 4 shows the exergy efficiency of the two-stage power generation system varying with the evaporation temperature (T1, T6) of the organic working fluid in the high and low temperature systems when the heat source temperature is 130 °C and the mass split ratio of the high temperature system preheater is 0.65. It can be seen that as the evaporation temperatures of the working fluid in the high-temperature system and the low-temperature system increases, the exergy efficiency of the system has a maximum value, that is, at a certain heat source temperature, the exergy efficiency of the system is affected by both the high and low temperature systems. When the evaporation temperatures of the organic working fluid in the high and low temperature systems are 100.1 °C and 69.8 °C, respectively, the system reaches a highest exergy efficiency of 37.0%. Compared with the single-stage system and regenerator system under the same parameters, the exergy efficiency of the system is increased by 10.1% and 3.8%, so the two stage system exergy efficiency is higher, which is consistent with Safarian S et al. [13] and Kaşka Ö et al. [14]. The increase in exergy efficiency is mainly due to the more optimized matching of the temperature curves of the hot and cold fluids in the heater and condenser of the two-stage system, which reduces the loss of available energy of the components, thereby increasing the exergy efficiency of the system.

**Figure 5:**
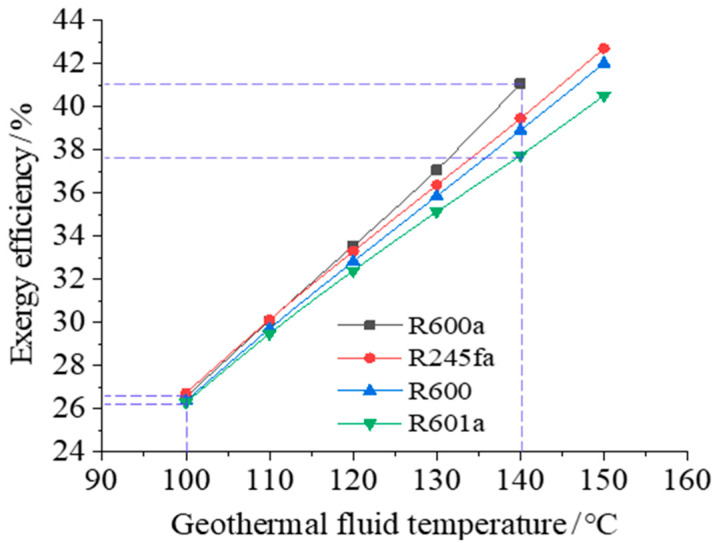
Exergy efficiency of the system changes with the temperature of the heat source.

The exergy efficiency of system for the four different organic working fluids shown in Table 1 and the maximum exergy efficiency of the system at different heat source temperatures are further analyzed when the heat source temperature is in the range of 100 °C~150 °C. The results are shown in Figure 5. It can be obtained from the figure that the system exergy efficiency of different working fluids shows an increasing trend with the increase of the heat source temperature: when the heat source is at a higher temperature, energy is utilized more efficiently, and the system exergy efficiency is higher. Among the four selected working fluids, the system exergy efficiency is the highest for R600a, this conclusion is consistent with the results of E.ÖZDEMİR et al. [9]. However, it is only suitable for heat source temperature below 140 °C, while other working fluids can be applied to systems with higher heat source temperatures.

With high heat source temperature, the exergy efficiencies of the system for different working fluids are quite different, indicating that the selection of suitable working fluid can improve energy utilization. For example, when the heat source temperature is 140 °C and the working fluids of the two-cycle system are R600a and R601a, the exergy efficiencies of the system are 41.1% and 37.7%, respectively, with a relatively large difference. When the heat source temperature is 100 °C, the corresponding system exergy efficiencies are respectively 26.6% and 26.3%, respectively, with a very small difference.

**Figure 6:**
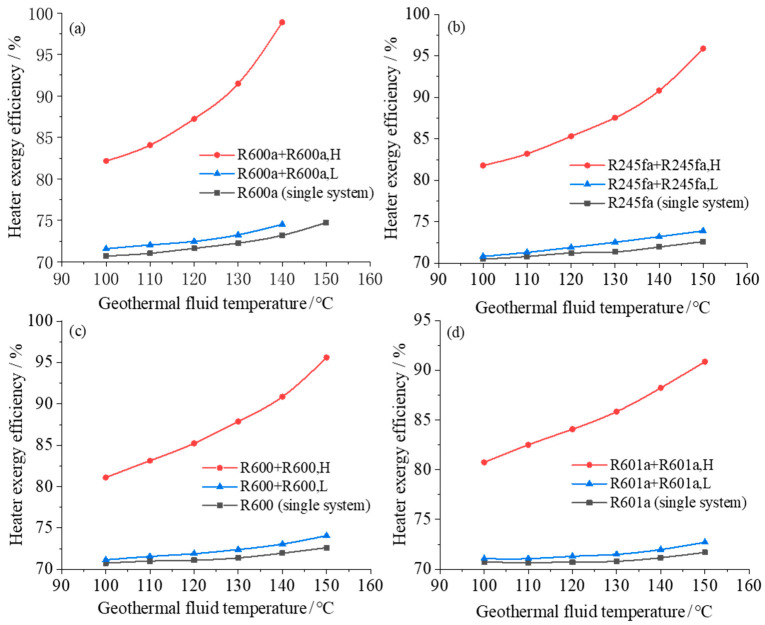
Heater exergy efficiency varies with geothermal fluid temperature in both single system and dual system with different working fluids: (**a**) R600a; (**b**) R245fa; (**c**) R600; (**d**) R601a.

**Figure 7:**
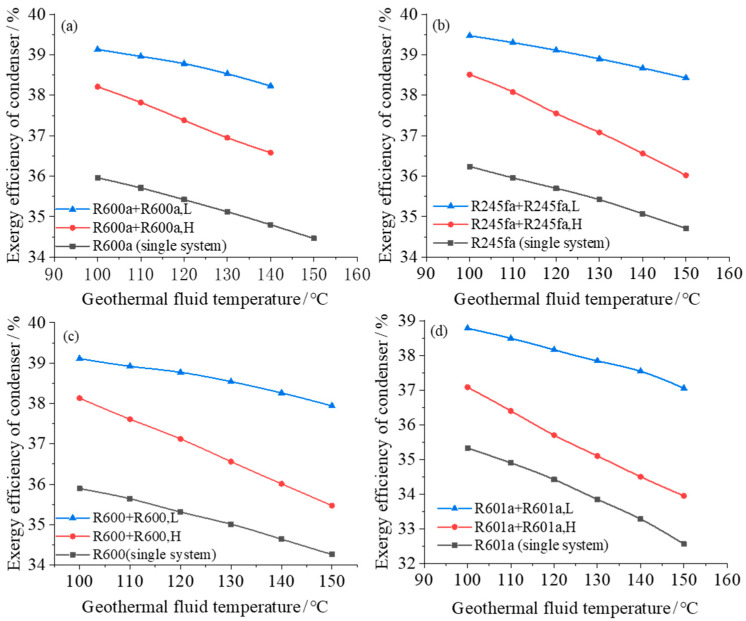
Exergy efficiency of condenser varies with geothermal fluid temperature in both single system and dual system with different working fluids: (**a**) R600a; (**b**) R245fa; (**c**) R600; (**d**) R601a.

The heater (including evaporator and preheater) and condenser are associated with large exergy loss in ORC power generation system. Therefore, the exergy efficiency of the equipment in the two-stage and single-stage ORC system is further analyzed and compared. Figure 6 and Figure 7 show how the exergy efficiencies of the heater (evaporator and preheater) and condenser in two-stage/single-stage ORC system vary with the heat source temperature under different geothermal fluid temperatures. It can be seen from the Figure 6 and Figure 7 that as the temperature of the geothermal fluid increases, the exergy efficiencies of the heater and the condenser have opposite trend: the exergy efficiency of the heater increases with the heat source temperature while the exergy efficiency of the condenser decreases with the heat source temperature. As the heat source temperature increases, the exergy efficiency of the heater of the high-temperature system increases and the exergy efficiency of the condenser in the low-temperature system decreases.

The exergy efficiency of the heater in the high-temperature system of the two-stage system is significantly larger than that in the single-stage system. Since the thermal fluid cooling process matches the working fluid heating-evaporation process more appropriately, the average temperature difference between the cold and hot fluids gradually decreases, and the irreversible loss is gradually reduced. When the heat source temperature is 140 °C and the working fluid is R600a, the exergy efficiencies of the heater in high temperature system, low temperature system and single-stage system are 98.8%, 74.5% and 73.2%, respectively. Compared with the single-stage system, the average heater exergy efficiency of the two-stage system is increased by ~13.4%, indicating that the two-stage system effectively improves the exergy efficiency of the equipment.

The exergy efficiency of the condenser in the two-stage system decreases with the increase of the heat source temperature, and the reduction is more rapid for the condenser in the low-temperature system. The main reason is that as the heat source temperature increases, the temperature of the geothermal fluid entering the condenser increases, so that the temperature difference between the cold and hot fluids during the condensation process increases and consequently the energy loss is larger. Therefore, the exergy efficiency decreases as the heat source temperature increases. For a two-stage cycle system with R600a, when the heat source temperature increases from 110 to 140 °C, the arithmetic average of the heater exergy efficiency increases by 8.4%, while the condenser exergy efficiency decreases by 1.9%. The equipment exergy analysis shows that the system exergy efficiency increases with the increase of heat source temperature.

### 3.2. Analysis of Preheater Split Ratio

The net output power of a power generation system is mainly related to the enthalpy drop of the expander and the mass flow of working fluid. In the calculation conditions in this paper, when the geothermal fluid and the condensing temperature are fixed, the optimal evaporation temperature of the system is fixed, meaning the enthalpy drop of the expander is fixed. The main factor affecting the flow rate of working fluid is the pinch point temperature. Therefore, when the pinch point temperature is a constant, the net output power of the power station is uniquely determined. With reasonable split ratio of the geothermal fluid in the high temperature and low temperature systems, the net output power is not affected by the split ratio, but only affected by pinch temperature difference.

**Figure 8:**
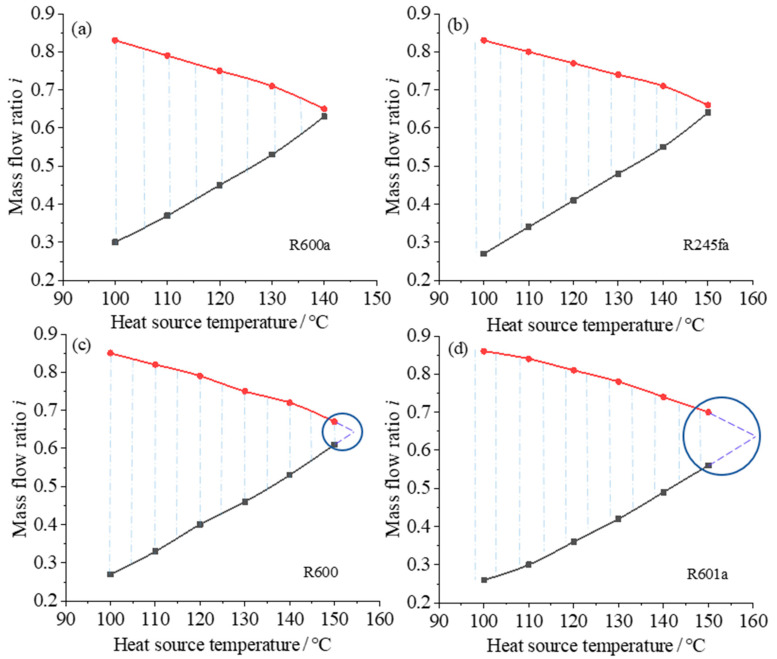
Relationship between split ratio of preheater and heat source temperature for different working fluids: (**a**) R600a + R600a; (**b**) R245fa + R245fa; (**c**) R600 + R600; (**d**) R601a + R601a.

**Figure 9:**
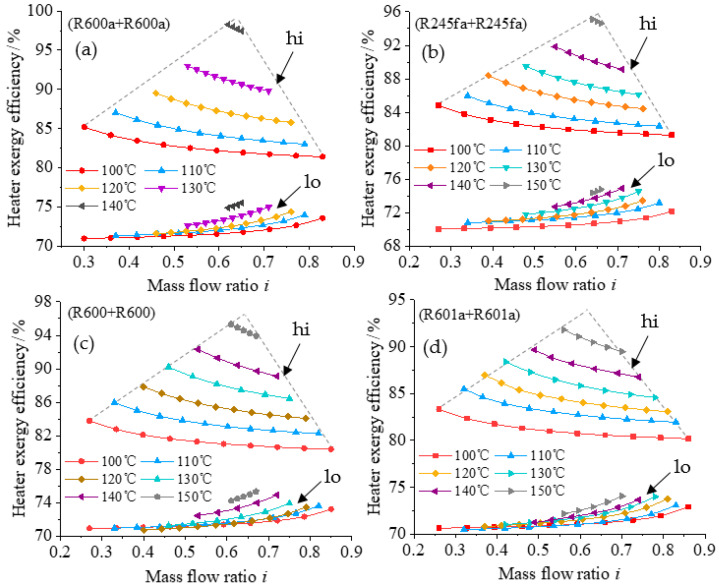
Relationship between heater exergy efficiency and mass split ratio for different working fluids: (**a**) R600a + R600a; (**b**) R245fa + R245fa; (**c**) R600 + R600; (**d**) R601a + R601a.

The reasonability of the split ratio is determined by parameters such as the exergy efficiency of the preheater and the temperature of the geothermal fluid at the outlet of the preheater. The variation among different parameters are analyzed under the condition when the exergy efficiency of the system is maximized and the heat source temperature is in the range of 100~150 °C. Figure 8 shows the variation of the range of split ratio with different heat source temperature for the four different working fluids, and Figure 9 shows the variation of the heater exergy efficiency with different split ratio.

It can be seen from Figure 8 that when the geothermal temperature increases, the range of the split ratio (shown by the blue dashed lines) becomes smaller, and the trend of the split ratio for different working fluids all presents an isosceles triangle shape. The split ratio chart shows that the applicable heat source temperature ranges for different working fluids are different: the split ratio range is large at low temperature, and the split ratio range becomes smaller at higher temperature until the working fluid is no longer applicable. For example, R600a is suitable for low-temperature heat sources below 140 °C, and R601a is suitable for higher heat source temperatures, the suitable heat source temperature of R245fa and R600 is between them. This conclusion is consistent with the previous analysis. Since the maximum heat source temperature in the current analysis is 150 °C, the predicted applicable range is shown by the dashed line in Figure 8c,d. The main reason for the change of the split ratio range with the heat source temperature is that when the heat source temperature is low, the working fluid flow rate is relatively reduced, so the heat required for heating in the preheater is correspondingly reduced, resulting in a decrease of the flow distribution for the heat source. The applicable range of different working fluids at high temperatures is mainly determined by the physical properties of the working fluids.

In summary, when R600a is selected for the two-stage cycle system, the system exergy efficiency is higher, but it is not suitable for high-temperature heat sources. R601a is suitable for high temperature heat sources, but the exergy efficiency of the system is relatively low. Considering the system efficiency and heat source temperature, the performance and applicable temperature range of R245 and R600 are between the working fluids of R601a and R600a. Therefore, it is necessary to select the appropriate working fluid according to the temperature of the heat source. 

Figure 9 shows the relationship between the exergy efficiency of the heater and the split ratio in the high temperature and low temperature systems. It can be seen from the figure that the exergy efficiency of the heater increases with the increase of the heat source temperature. The exergy efficiency of the heater in the high temperature system increases at a higher rate than that in the low temperature system. For example, when the split ratio is 0.65 and the heat source temperature increases from 100 to 140 °C, the heater exergy efficiency of the high-temperature system increases by 15.5% when R600a is the working fluid and by 6.9% when R601a is the working fluid. This is consistent with previous analysis that the changes of exergy efficiencies of the main components of the system have the greatest impact on the system exergy efficiency.

The heater exergy efficiency of the high temperature system presents a triangular shape with the change of heat source temperature and split ratio, as shown by the dotted line in Figure 9. The overall trend is the same for different conditions, although the shape is slightly different because the rate of change with temperature is different. This is mainly because the split ratio and the heater exergy efficiency in the high temperature system vary greatly with the heat source temperature. However, the heater exergy efficiency in the low temperature system has small variation and does not present the above trend.

## 4. Conclusions

An exergy analysis was performed to study the two-stage ORC power generation system with a geothermal fluid temperature of between 100 and 150 °C. The main conclusions are listed as follows:

1. The exergy efficiency of the two-stage system is larger than that of the single-stage system, and there is a maximum value when the cold and heat source parameters are fixed. The exergy efficiency of the two-stage system is increased by 9.4% compared with the single-stage system when the heat source temperature is 130 °C.
2. Among the four selected organic working fluids, R600a has the highest system exergy efficiency, which is more obvious as the heat source temperature increases. However, R600a is only suitable for heat source temperature below 140 °C. Other working fluids can be applied to power generation systems with higher heat source temperatures. Appropriate organic working fluids need to be selected according to the heat source temperature.
3. As the temperature of the geothermal fluid increases, the exergy efficiency of heaters and condensers in the two-stage system show opposite trends. As the heat source temperature increases, the heater exergy efficiency increases but the condenser exergy efficiency decreases. In addition, the exergy efficiency of the evaporator of the high-temperature system is greatly increased compared with the single-stage system, which is the main factor for the increase of the exergy efficiency of the system.
4. The mass split ratio for preheaters of the two-stage cycle system is related to parameters such as working fluid type and heat source temperature. The split ratio range is larger at lower temperature and smaller at higher temperature. The variation trend of the split ratio presents an isosceles triangle shape. Different working fluids are suitable for different heat source temperature ranges. R600a working fluid is suitable for low temperature heat sources below 140 °C. Therefore, it is necessary to determine the appropriate working fluid and split ratio according to the heat source parameters.

## Figures and Tables

**Table 1:** Selection of working fluids and physical properties.

Working Fluid	Category	Standard Boiling Point/°C	Critical Temperature/°C	Critical Pressure /MPa	Security Level
R600a	Hydrocarbon	−11.7	134.7	3.64	A3
R245fa	Hydrofluorocarbon	15.1	154.1	4.43	B1
R600	Hydrocarbon	−0.5	152.0	3.8	A3
R601a	Hydrocarbon	27.8	187.4	3.39	A3

**Table 2:** Main parameters.

Parameter	Value
*m*_g_	1 kg/s
*η*_pu_	72%
Δ*t*_hi_	6 °C
Δ*t*_lo_	5 °C
*T*_0_	292 K
*t*_3_/t_7_	30 °C

## Data Availability

The data supporting the findings of this study are available from the corresponding author (Z.M.) upon reasonable request.

## Nomenclature

*List of symbols*	
${\bar{c}}_{p}$	average specific heat of geothermal fluid [kJ/kg·K]
*h*	enthalpy [kJ/kg]
*m*	mass flow rate [kg/s]
*p*	Pressure [kPa]
*s*	Entropy [kJ/kg·K]
*t*	Centigrade [°C]
*T*	Kelvin temperature [K]
*v*	mass volume [m^3^/kg]
*W*	power [kW]
*i, j*	flow rate ratio
*Abbreviations*	
ORC	organic Rankine cycle
Greek symbols	
*η*	efficiency
Δ*t*	Pinch temperature
*Subscripts*	
co	condenser
cw	cooling water
co	condenser
cw	cooling water
ev	evaporator
ex	exergy
exp	expander
hi	high temperature system
in	cooling water inlet
g	geothermal fluid
ga	gain
lo	low temperature system
net	net power
o	environment
out	Cooling water outlet
pa	pay
ph	preheater
pu	pump
wf	working fluid
a,b,c,d,1,2,3,4,5,7,9,10	Corresponding to the number position in Figure 1 and Figure 2
